# Molecular Spectroscopy Evidence of 1,3,5-Tris(4-carboxyphenyl)benzene Binding to DNA: Anticancer Potential along with the Comparative Binding Profile of Intercalation via Modeling Studies

**DOI:** 10.3390/cells12081120

**Published:** 2023-04-10

**Authors:** Tanveer A. Wani, Seema Zargar

**Affiliations:** 1 Department of Pharmaceutical Chemistry, College of Pharmacy, King Saud University, P.O. Box 2457, Riyadh 11451, Saudi Arabia; 2 Department of Biochemistry, College of Science, King Saud University, P.O. Box 22452, Riyadh 11451, Saudi Arabia; szargar@ksu.edu.sa

**Keywords:** DNA-ligand interaction, molecular docking, molecular dynamic simulation, groove binding, cancer

## Abstract

One of medicinal chemistry’s top priorities is the discovery of new molecules with anticancer potential. Compounds that interact with DNA are an intriguing family of chemotherapeutic medications used to treat cancer. Studies in this area have uncovered a plethora of potential anticancer medicines, such as groove binding, alkylating, and intercalator compounds. The anticancer activity of DNA intercalators (molecules that intercalate between DNA base pairs) has drawn special interest. The current study investigated the promising anticancer drug 1,3,5-Tris(4-carboxyphenyl)benzene (H3BTB) against breast and cervical cancer cell lines. In addition, 1,3,5-Tris(4-carboxyphenyl)benzene binds to DNA by groove binding. The binding of H3BTB to DNA was found to be significant which unwinds the DNA helix. Considerable electrostatic and non-electrostatic contributions were present in the binding’s free energy. The cytotoxic potential of H3BTB is effectively demonstrated by the computational study outcomes, which include molecular docking and molecular dynamics (MD) simulations. The minor groove binding for the H3BTB–DNA complex is supported by molecular docking research. This study will promote empirical investigation into the synthesis of metallic and non-metallic H3BTB derivatives and their potential use as bioactive molecules for the treatment of cancer.

## 1. Introduction

Cancer is an intricate illness that manifests itself in the unchecked development and systemic metastasis of aberrant cells. As one of the leading killers across the world, it presents a formidable obstacle for the medical community [1]. Conventional cancer treatments, such as chemotherapy and radiation therapy, are inefficient and have serious side effects [2]. As a result, there is a growing interest in developing novel and effective anticancer drugs. One technique for developing new anticancer medications is to target particular proteins involved in the cancer pathway. The abnormal function of deoxyribonucleic acid (DNA) is mostly responsible for cancer. Drugs are supposed to destroy or reduce the size of cancer cells [3]. Furthermore, malignant cells’ DNA replicates by duplicating itself. The most efficient anticancer treatments should be able to block this replication by blocking cancer cells’ DNA, but they typically have major side effects on healthy cells as well [4]. As a result, non-reversible drug molecule binding to DNA is dangerous, whereas non-covalent reversible drug molecule binding is substantially safer and has the least cytotoxic effects on healthy cells [5]. As a result, it is still important to develop chemical compounds that can reversibly connect to DNA and inhibit cancer while causing little or no harm to healthy cells. In vitro compound–DNA binding studies using non-covalent interactions such as electrostatic interactions, groove binding, and intercalation [6] are extremely informative and useful for investigating the anticancer potential of a drug-like molecule in terms of binding modes and binding parameters [7,8,9]. Moreover, caspase-3, p53, and NF-κB have been identified as potential anticancer therapeutic targets [10]. Caspase-3 is a cysteine protease that is required for the apoptotic process. p53 is a tumor suppressor protein that affects the cell cycle and DNA repair pathways. NF-κB is a transcription factor that regulates immunological response and inflammation. The deregulation of these proteins has been associated with tumor growth and progression [11,12,13].

The potential of new drug molecules to inhibit cancer growth that target DNA, caspase-3, p53, and NF-κB has been a subject of continuous research. The apoptosis-related protease caspase-3 has been proposed as a therapeutic target for the treatment of cancer [14]. The P53 protein is known to commonly mutate when it is present in cancer cells [15]. Restoring the function of p53 has been shown to prevent the formation of cancer cells [16], which supports the hypothesis that p53 has a potential role in the control of cell survival and inflammation. NF-κB is important because it regulates genes involved in cell growth and survival [17]. Heterocyclic compounds are an important chemical class of drugs in medicinal chemistry and the rational design of novel medications [18].

For a very long time, chemistry has made extensive use of molecules with C3 symmetry and compounds with two-fold rotational symmetry, particularly in asymmetric catalysis and chiral recognition. C3-molecules have a structural moiety that is repeated three times around a rotational axis, just like C3-organisms and objects, and they can have an acyclic, exocyclic, macrocyclic, or bicyclic topology. The existence of a second rotational axis that goes in the opposite direction can occasionally complicate this C3-symmetry [19]. These substances are referred to as D3-symmetric. However, relatively few commercially available drugs exhibit C3-symmetry, in contrast to C2-drugs whose structures typically correspond to the homodimeric status of their therapeutic target, such as anticancer effects and DNA binding properties [20]. Here we are focusing on H3BTB, also known as 1,3,5-tris(4-carboxyphenyl)benzene for its anticancer potential by targeting DNA.

1,3,5-Tris(4-carboxyphenyl)benzene (H3BTB) is an aromatic hydrocarbon that belongs to the class of compounds known as triphenylbenzenes. It is characterized by the presence of three phenyl rings that are connected by a central benzene ring. Moreover, it is a symmetrical molecule that has three carboxylic acid groups attached to each phenyl ring, which is what gives it its acidity and allows it to be soluble in water [21]. The molecule 1,3,5-tris(4-carboxyphenyl)benzene (H3BTB), which has the C3-symmetry and exists in only two dimensions, forms a self-assembled monolayer (SAM) on a wide range of substrates. Moreover, it is also known as a tritopic bridging ligand that makes it feasible to functionalize metal organic frameworks (MOFs) based on polyoxometalates for probable applications in gas storage, gas separation, and catalysis [22,23].

**Figure 1:**
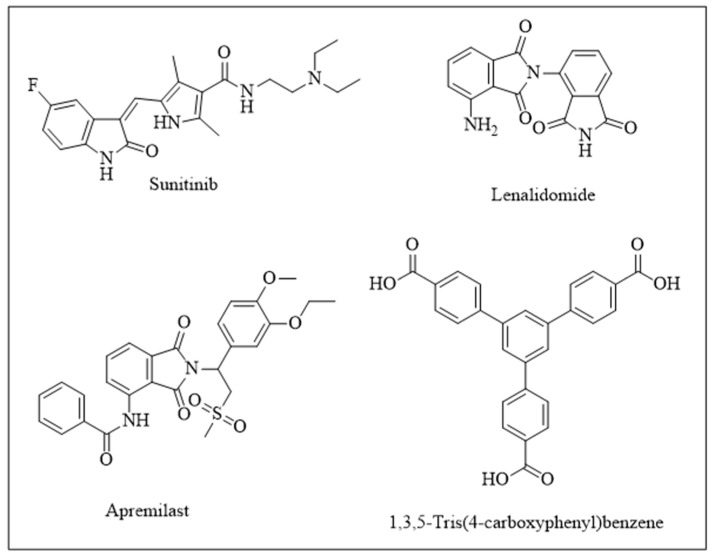
Illustration of compounds possessing carboxy phenyl benzene moiety [17,18,19,20].

This H3BTB is also a component of metal organic frameworks (MOFs). MOFs are 3D-microporous materials that could be used in gas separation and adsorption processes [24,25]. In recent years, H3BTB has been employed as a linker to create MOFs with incredibly large surface areas, such as MOF-177 (5000 m^2^/g), a hydrogen-absorbing substance with an incredibly high hydrogen storage capacity of 7.5% at 77K. The compounds with a carboxy phenyl benzene moiety are shown in Figure 1.

Along with experimental studies, computational approaches can help find new anticancer drugs. DFT is commonly used to examine molecules’ electronic structure and characteristics [26]. Molecular docking and MD simulations predict ligand-protein binding affinity and stability, respectively, using computational methods. We investigated the anticancer potential of 1,3,5-tris(4-carboxyphenyl)benzene using experimental and computational methods. This study tested the compound’s cytotoxicity against cancer cells using several cell lines. We determined the compound’s electrical characteristics using DFT. Molecular docking and MD simulations examined the compound’s binding affinity and stability with caspase-3, p53, and NF-κB. These chemicals interact with proteins via hydrogen bonding and electrostatic interactions due to carboxylic acid groups. Carboxyphenyl moieties conjugated to a central benzene ring improve molecular stability and stiffness. These properties make compounds containing carboxyphenyl moieties promising candidates for anticancer therapy.

## 2. Experimental

### 2.1. Cell Viability Assay

The anticancer potential of 1,3,5-Tris(4-carboxyphenyl)benzene was investigated against two cell lines, i.e., human breast cancer cell lines (MDA-MB231; ATTC: HTB-26™ and MCF-7 cell line; and ATTC: HTB-22™) and one human cervical cancer cell line (HeLa; ATTC: CRM-CCL-2™). The effect was also observed against normal cell lines, i.e., African green monkey kidney (Vero) cells (ATTC: CCL-81™). The cell viability assay was accomplished by slightly modifying Mosmann’s 1983 and Nikš and Otto’s 1990 methods [27,28]. To each well, 10 × 10^4^ cells were seeded and 10 μL of compound (and also doxorubicin and cisplatin) at the final concentration of 50 µM was treated with cells and then plates were kept in an incubator at 37 °C and 5% CO_2_. After 24 h, 10 μL of MTT reagent was added followed by the incubation of 4 h at 37 °C. The stopping reagent, i.e., 10% sodium dodecyl sulfate, was added and optical density was observed at 575 nm along with the background absorbance at 625 nm. The percentage growth inhibition values and inhibitory concentration values (IC_50_) were calculated by using GraphPad Prism Software version 5.0 [28].

### 2.2. Spectrophotometric DNA Binding Analysis

UV-visible spectrophotometric measurements were performed at room temperature to observe the binding interaction of 1,3,5-Tris(4-carboxyphenyl)benzene with DNA. 1,3,5-Tris(4-carboxyphenyl)benzene was prepared in 10% DMSO. Lyophilized Herring sperm DNA (Sigma Aldrich, Burlington, MA, USA) was weighed and its stock solution was made by dissolving 5 mg of powder in 10 mL of distilled water. The ratio of absorbance at 260 and 280 nm was used to determine purity. The ratio was between 1.6 and 1.9, indicating that DNA was pure enough for testing. In the absence and presence of different concentrations of HS–DNA (40 μM, 80 μM, 120 μM, 160 μM, 200 μM, and 240 μM), the experiment was conducted [29,30]. After 30 min in the dark at room temperature, a FLUOstar Omega microplate reader recorded UV absorption spectra (BMG Labtech, Ortenberg, Germany).

### 2.3. Computational Investigations

#### 2.3.1. Density Functional Theory Calculations

The structural geometry of the 1,3,5-Tris(4-carboxyphenyl)benzene was optimized and frequency calculations were performed using the Gaussian 09W program [31]. This was done to ensure that the structural parameters of the H3BTB were stable and accurate [32]. In addition, frontier molecular orbitals and local and global reactivity descriptors [33] were calculated to investigate the electronic density of the compound. The reactivity profile of the H3BTB was characterized in terms of electron density, which resulted in high ionization potential and chemical softness. The density functional theory (DFT) calculations were performed using the B3LYP functional correlation and 6-31G* as the basis set [34]. The 6-31G* basis set is a combination of two basis sets: the 6-31G and the 3-21G basis sets. It provides a good balance between accuracy and computational efficiency and includes additional diffuse functions that are important for representing the electron density in the outer regions of a molecule. The selected basis set is composed of primitive and Gaussian-type orbitals [35], which provide accurate and efficient representations of the electronic properties of the compounds [36]. The various analytical metrics, including global and local reactivity descriptors, were derived by analyzing log files in GaussView 06 [37]. This helped in determining the electronic properties of the compounds and their suitability as potential ligands.

#### 2.3.2. Molecular Docking Studies

The investigation of non-covalent interactions is essential in determining the inhibitory potential of the compounds [38]. In this study, 1,3,5-Tris(4-carboxyphenyl)benzene was subjected to molecular docking against multiple anti-cancer proteins including caspase-3, NF-κB, P53, and DNA. The targeted proteins were retrieved from the Protein Data Bank (PDB ID: 3DEI, 1NFI, 3DCY, and 127D, respectively, www.rcsb.com (accessed on 15 Feburary 2023)). The targeted macromolecules were initially prepared for the molecular docking process using MGL tools [39]. This preparation process involved the removal of water and heteroatoms, the addition of polar hydrogen atoms, and the incorporation of Gasteiger charges. The protonation state of the proteins is important in determining the binding pattern of the ligand inside the active pocket of the targeted enzyme. Missing residues were fixed using MGL tools to ensure an accurate representation of the proteins. After preparation, all proteins were retrieved in the desired format for docking. The next step was the preparation of the 1,3,5-Tris(4-carboxyphenyl)benzene for the docking process, which involved retrieving the optimized ligand from the density functional theory calculations and preparing the database for the docking process. 1,3,5-Tris(4-carboxyphenyl)benzene was saved in the pdbqt format for docking. The AutoDock Vina docking [40] wizard was employed for molecular docking after the preparation of the protein and ligand database. The grid box was expanded to encompass the x, y, and z centers of caspase-3 (−46.790, 15.0200, and −21.901), NF-κB (−10.249, 48.903, and 6.706), p53 (30.483, 32.901, and −2.936), and the entire DNA, which were selected for docking, respectively. The number of modes was set to 100 poses to eliminate any false positives and generate only reliable docking poses. Finally, the docking output files were analyzed, and the top-ranked conformations were subjected to further QM/MM analysis.

The validation of the docking protocol is a crucial step in determining its reliability. To validate the docking process, the redocking of the co-crystal ligand (standard inhibitor) was performed, and the RMSD value of fewer than 2 angstroms between the native and regenerated pose was considered a validated docking procedure [39].

### 2.4. Molecular Dynamics Simulations

Molecular dynamics simulations were run using the Desmond program on the protein–ligand complex that was obtained from molecular docking. The simulation of each complex took place in the TIP3P solvent model for the duration of 50 ns [41]. Counter sodium chloride (NaCl) ions with a concentration of 0.15 M were added to neutralize the system. The forcefield used for the simulation was the OPLS3 (optimized potential for liquid simulation 3) [42], which integrated the motion of atoms under periodic boundary conditions. A preliminary energy minimization approach was applied for 2000 steps to avoid any clashing of atoms. At a temperature of 300 kelvin and a pressure of 1.01 bar, the system was brought to a state of equilibrium in an isothermal and isobaric (NPT) [43] ensemble [44]. To consider short-range van der Waals interactions, a cutoff distance of 10 angstroms was employed. During the simulation, a Nose-Hoover thermostat in conjunction with a Martyna–Tobias–Klein barostat was utilized to keep the pressure and temperature stable [45]. When integrating the motion equations, a time step of 2 fs was utilized for the calculation. It took a total of 50 ns to complete the production run, during which time the simulated trajectories were saved at intervals of 50 picoseconds. The particle mesh Ewald approach was utilized to conduct an accurate and trustworthy investigation into the electrostatic interactions [46]. To analyze the simulated trajectories followed by the protein-ligand complexes, the Desmond simulation interaction diagram procedure was utilized.

## 3. Result and Discussions

### 3.1. In Vitro Cytotoxic Activity

**Figure 2:**
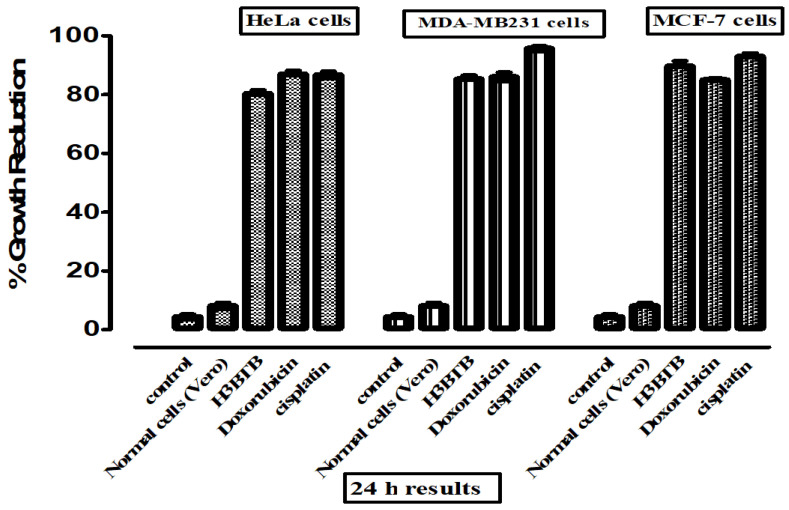
The graphical presentation of % growth reduction exhibited by H3BTB against normal cells (Vero) and three cancerous cell lines (HeLa, MDA-MB231, and MCF-7) after 24 h.

The compound H3BTB was tested using an in-vitro colorimetric MTT assay against three cancer cells lines; specifically, HeLa (human cervical cancer cell line), MCF-7, and MDA-MB-231 (human breast carcinoma cancer cell line) [47,48]. The compound was also tested on normal cells (Vero) and total activity control was also used during the experiment. In this cytotoxic evaluation, doxorubicin and Cisplatin were used as reference compounds. The results of the % growth reduction are given in Figure 2 below, and GI_50_ is presented in Table 1.

As shown in Table 1, the H3BTB was found to be most active against MDA-MB231 and MCF-7 with GI_50_ values of 7.62 ± 0.91 μM and 9.03 ± 0.18 μM, respectively. On the other hand, this compound was identified as the least active agent against the HeLa cell line. When the activity was compared with the two positive controls it was found that the selected compound has good anti-cancer potential and can be used as the lead pharmacophore for the synthesis of more potential anti-cancer compounds. The anti-cancer activity was further supported by the DNA-binding studies.

### 3.2. Spectroscopic Studies for 1,3,5-Tris(4-carboxyphenyl)benzene–DNA Binding

**Figure 3:**
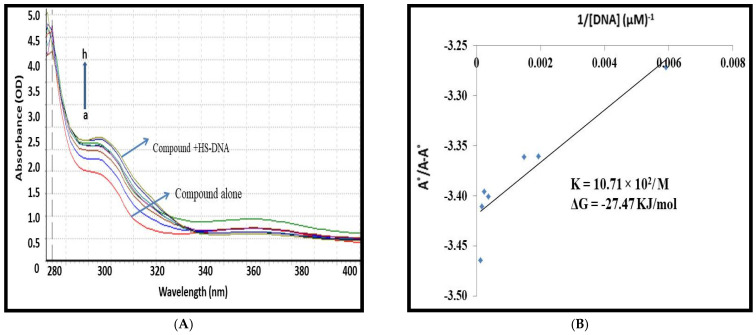
(**A**) UV-visible spectral responses of H3BTB in the absence and presence of different HS-DNA concentrations as mentioned before. The arrowhead indicates the increasing concentration of DNA. (**B**) Ao − A/Ao vs. 1/[DNA] graph for binding constant calculation.

UV-visible spectroscopy was used to study the changes in the UV-visible spectrum of H3BTB in the presence of double-stranded HS-DNA, in addition to molecular docking to identify the DNA-binding interaction. A ligand molecule’s spectral alterations may produce a hyperchromic or hypochromic effect in the presence of DNA, with or without a peak shift towards a longer or shorter wavelength. The decrease or increase in peak intensity along with the shift in the red and blue wavelengths suggested that a compound and DNA intercalated through intercalative interaction; the increase in peak intensity suggested that the cationic part of the ligand may have electrostatically bound to the anionic part (PO_4_^3−^) of the DNA backbone; and the spectral variation in terms of hypo- or hyperchromic effect with no or minimal shift in peak position (6–8 nm) suggested a weaker molecule [49,50,51]. The H3BTB and HS-DNA spectral profiles each displayed a single peak at 328 nm and 260 nm, respectively. As shown in Figure 3, the change in the H3BTB spectrum was detected as a progressive increase in absorbance following treatment with different DNA concentrations. Using the absorbance values of H3BTB before and after the formation of the compound-DNA adduct, the following equation [52] was used to calculate the percentage increase in peak intensity.

(1) $$H\%=\frac{A_{ligand}-A_{ligand-DNA\;adduct}}{A_{ligand}}\times 100$$

Figure 3 presents the H3BTB absorption spectra measured with HS-DNA. It was revealed that the concentration of HS-DNA caused an increase in the absorbance of the sample, which was accompanied by a blue shift of 10 nm and a hyperchromic effect of 59.3%. Earlier research found that the interaction of ligands with DNA can result in the formation of complexes and that these complexes can cause a shift in the system’s absorbance in either the red or blue direction [53]. Since there was a rise in absorbance, it can be concluded that intercalative binding between HS-DNA and H3BTB did not take place. On the other hand, it can be concluded that there was minor groove binding between H3BTB and HS-DNA. These findings are consistent with those found in earlier research of HS-DNA in conjunction with other medications [54].

The binding constant (*Kb*) was found by comparing the absorbance of selected H3BTB before (*A*_0_) and after (*A*) adding DNA to the Hildebrand Equation (2). This was used to figure out the Gibbs free energy change (Δ*G*) from the Van’t Hoff Equation (3) [53,54,55,56].

(2) $$\frac{A_{0}}{A-A_{0}}=\frac{\epsilon_{G}}{\left(\epsilon_{H-G}-\epsilon_{G}\right)}+\frac{\epsilon_{G}}{\left(\epsilon_{H-G}-\epsilon_{G}\right)}\;1/K_{b}\left[DNA\right]$$

Δ*G* = −*RT lnK_b_* (3)

The value of the binding constant was calculated using the intercept to slope ratio, which was obtained from the plot of *A*_0_/*A* − *A*_0_ vs. 1/[DNA] shown in Figure 3. It was determined that the values for *Kb* (M^−1^) and Δ*G* (kJ.mol^−1^) are 10.71 · 10^2^/M and −27.47 kJ/mol, respectively.

### 3.3. Density Functional Theory Calculations

The structural geometry of the compound was optimized, and frequency calculations were performed using the B3LYP/6-31G* level of theory. The structural geometry of H3BTB was optimized to the steepest energy gradient with no imaginary frequency. In addition, the electronic properties of H3BTB were also evaluated. Table 2 denotes the optimization and reactivity parameters of H3BTB.

**Figure 4:**
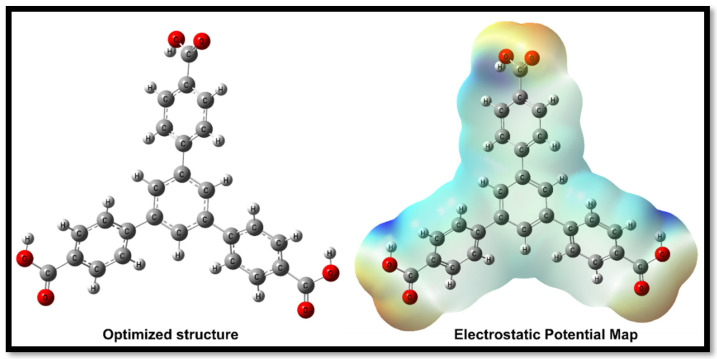
The optimized structure and ESP map potential of H3BTB.

The optimization energy of −1490.693076 Hartree is a measure of the stability of the compound in its optimized state. The polarizability of 326.515667 a.u indicates the compound’s ability to adjust its electron distribution in response to an external electric field. The dipole moment of 4.487465 debye represents the separation of positive and negative charges in the H3BTB, which contributes to its ability to interact with other molecules. The potential ionization of 0.257 eV is the energy required to remove an electron from the compound. The electron affinity of 0.0852 eV is the energy released when an electron is added to the compound. The electron-donating power of 0.096 and electron-accepting power of 0.267 describe the compound’s ability to donate or accept electrons, respectively. The electrophilicity (Δω±) of 0.362 represents the compound’s tendency to participate in electron transfer reactions and is a measure of its electrophilic or nucleophilic character. The electrostatic potential map highlights the regions of strong and weak electrostatic potential within the compound. The red-colored areas indicate a strong electron-attracting potential due to the presence of electronegative oxygen atoms, while the blue-colored regions represent a low electron-donating potential, most likely due to hydrogen atoms. These maps provide valuable insights into the electron distribution and reactivity of a molecule, allowing us to understand its properties and behavior. The optimized structure of H3BTB alongside the ESP map is provided in Figure 4.

According to the data, the EHOMO energy is −0.257 eV and the ELUMO energy is −0.0852 eV, resulting in an energy gap of 0.1718 eV. The chemical hardness is 0.086, which is a measure of the resistance of the molecule to chemical changes. The chemical potential is −0.171 and the electrophilicity index is 0.170; both indicating the tendency of the molecule to participate in chemical reactions. The chemical softness of the molecule is 5.821, which is a measure of the ease of chemical reactions. The electronegativity, a measure of the ability of the molecule to attract electrons, is 0.171. The global and local reactivity descriptor values for H3BTB are tabulated in Table 3.

**Figure 5:**
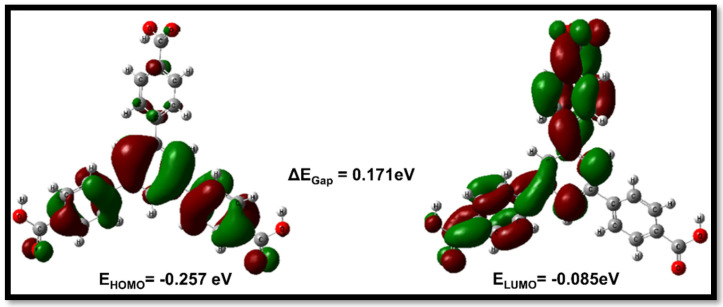
HOMO/LUMO orbitals of 1,3,5-Tris(4-carboxyphenyl)benzene.

The analysis revealed that the HOMO orbitals were localized to the aromatic rings, whereas the LUMO orbitals were delocalized throughout the molecule. This localization is particularly evident in the electronegative oxygen atom and phenyl rings, indicating the high electron affinity of the compound. On the other hand, the slight ionization potential is also visible as a result of the localization of the HOMO orbitals on the aromatic rings. Figure 5 provides a visual representation of the FMOs of H3BTB.

### 3.4. Molecular Docking Studies

In the current study, three cancer proteins (caspase-3, NF-κB, and p53) and the DNA intercalation property of H3BTB were evaluated. These proteins play a crucial role in cancer progression and development, making their inhibition a significant step in suppressing tumor growth. Caspase-3 is a key executioner of apoptosis (cell death) in response to cellular stress, NF-κB is a transcription factor that regulates the expression of genes involved in inflammation, cell survival, and proliferation, while p53 is known as the “guardian of the genome” as it regulates the cell cycle and acts as a tumor suppressor. Therefore, the evaluation of the interaction of H3BTB with these cancer proteins is of great significance in the field of cancer research. The results showed that H3BTB produced significant interactions with all three proteins and strongly intercalated the DNA molecule. Specifically, caspase-3 was substantially engaged in hydrophilic and hydrophobic interactions with the best docking score of −10.4 kcal/mol. These findings provide valuable insights into the potential of compound a as an anti-cancer agent and its mechanism of action. Table 4 denotes the docking scores and amino acids involved in binding interactions with H3BTB.

### 3.5. Interpretation of Molecular Interactions

**Figure 6:**
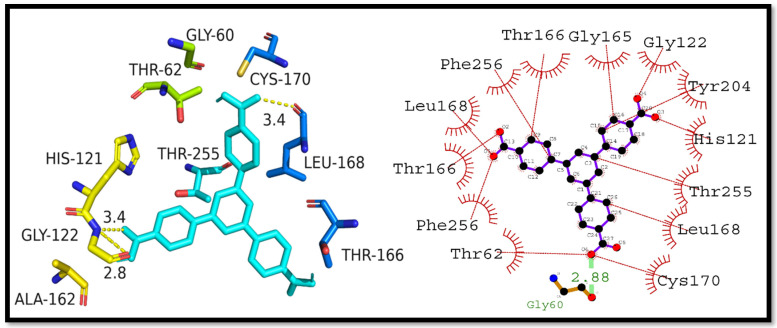
Presumed 2D and 3D binding mode of H3BTB with caspase-3.

In this study, the interaction between H3BTB and caspase-3 was evaluated through molecular docking simulations. The results showed that the complex formed between H3BTB and caspase-3 had a docking score of −10.4 kcal/mol, indicating a strong interaction between ligand and caspase-3. The hydrogen bonding between the compound and caspase-3 was observed to occur between the Gly60 residue of caspase-3 and the compound (Figure 6). The hydrogen bond length was calculated to be 2.88 angstroms. Additionally, several hydrophobic interactions were noted between caspase-3 residues, including Cys170, Leu168, Thr255, His121, Tyr204, Gly122, Gly165, Thr166, Phe256, Leu168, Thr166, Phe256, and Thr62, and the compound. These interactions further strengthened the binding between caspase-3 and H3BTB. Caspase-3 is a crucial protein involved in the process of apoptosis (programmed cell death) and plays a critical role in cancer progression. By forming a complex with caspase-3, H3BTB showed the potential to inhibit the protein’s function, thereby potentially suppressing the growth of cancer cells. These findings highlight the importance of understanding the interactions between compounds and proteins in the development of new cancer therapies.

**Figure 7:**
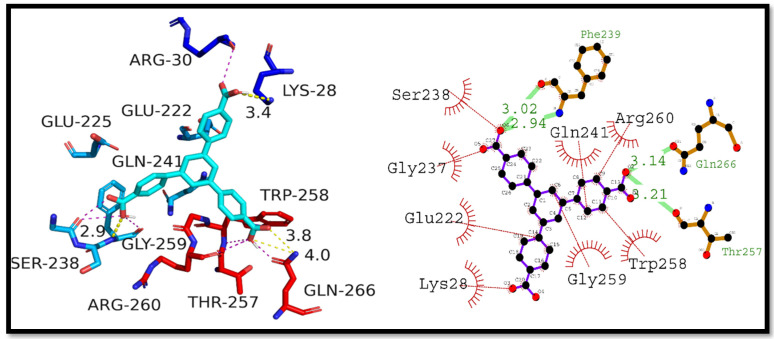
Presumed 2D and 3D interaction of H3BTB with NF-κB.

Similarly, the docking score for the NF-κB–H3BTB complex was determined to be −8.8 kcal/mol. The interactions were formed with Phe239 and Gln266 as the hydrogen bonding residues and hydrogen bond lengths of 3.02, 2.94, 3.14, and 3.21 angstroms. Hydrophobic interactions were observed with residues of Gly259, Trp258, Gln241, Arg260, Ser238, Gly237, Glu222, and Lys28 (Figure 7).

**Figure 8:**
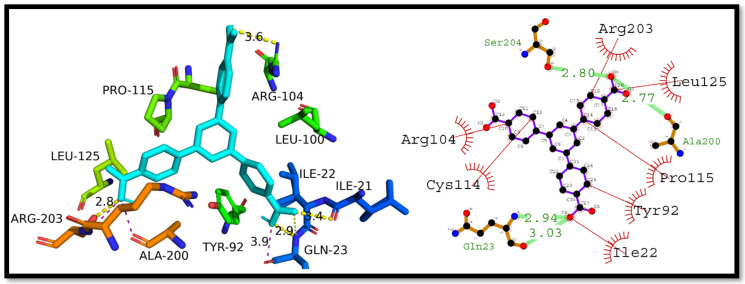
Presumed 2D and 3D interaction of H3BTB with p53.

The docking score for the P53–H3BTB complex was found to be −8.1 kcal/mol, formed hydrogen bonding with Gln23 and Ser204 residues, and hydrogen bond lengths of 2.94, 3.03, 2.80, and 2.77 angstroms. Hydrophobic interactions were observed with residues of Arg203, Leu125, Pro115, Tyr92, Ile22, Cys114, and Arg104. Figure 8 is illustrating the putative binding mode of H3BTB with p53.

### 3.6. Intercalation of Nucleic Acids

#### 3.6.1. Intercalation of DNA Molecules

**Figure 9:**
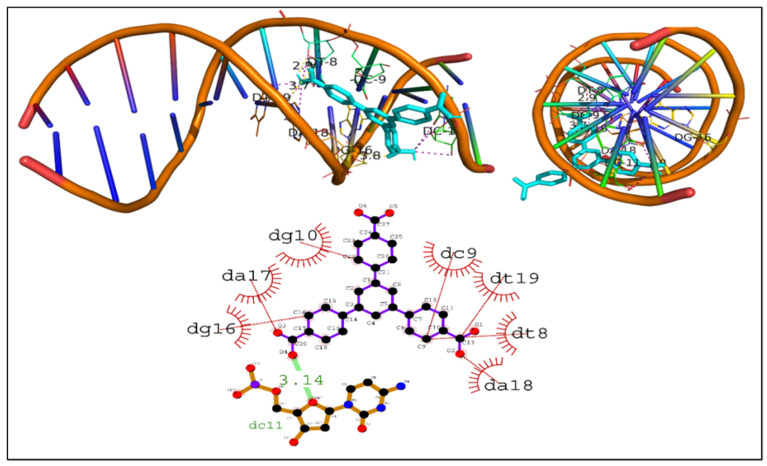
Intercalation of DNA groove by H3BTB.

In this study, it was observed that H3BTB exhibited strong intercalation into the major groove of the DNA molecule. The docking score for the DNA–1,3,5-Tris(4-carboxyphenyl)benzene complex was calculated to be −8.3 kcal/mol and was characterized by the presence of hydrogen bonding between the residue Dc11 and the compound with a hydrogen bond length of 3.14 angstroms. Furthermore, hydrophobic interactions were identified between H3BTB and residues Gd16, Da17, Dg10, Dc9, Dt19, Dt8, and Da18. These findings demonstrated the substantial interactions between H3BTB and the cancer proteins and DNA, and thus suggested its potential as a promising inhibitor of tumor growth. Figure 9 illustrates the intercalation of the DNA groove.

#### 3.6.2. Intercalation of RNA Molecule

**Figure 10:**
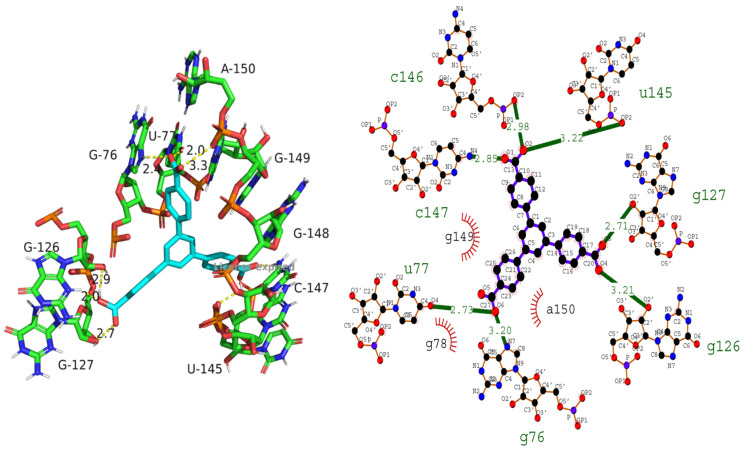
Intercalation of RNA groove by H3BTB.

The interaction between small molecules and nucleic acids plays an important role in various biological processes. Molecular docking is a widely used computational method to study these interactions and predict the binding mode and affinity of small molecules to nucleic acids. In this research, we used molecular docking to investigate the binding of H3BTB to RNA and interpret the obtained data. The binding site for compound a on RNA was found to be C146, C147, U77, U145, G127, G126, G76, and G78, with distances ranging from 2.73 to 3.22 Å. The H3BTB–RNA complex also formed additional hydrogen bonds with G149 and A150. The binding energy of the RNA–compound complex was found to be −9.5 kcal/mol. The binding site of H3BTB on RNA was found to be mainly comprised of pyrimidine bases (C and U) and purine bases (G and A). This suggests that the compound forms interactions with the nucleic acid bases through hydrogen bonding and hydrophobic interactions. The additional hydrogen bonds formed with G149 and A150 indicate that H3BTB may form specific interactions with these bases, which could play a role in the overall stability of the complex. The binding energy of −9.5 kcal/mol indicates a strong interaction between compound a and RNA. This suggests that compound a has a high affinity for RNA and may have potential as a therapeutic agent targeting RNA molecules. The interpretation of the binding site and binding energy data obtained from molecular docking provides valuable insights into the molecular interactions between H3BTB and RNA, which could aid in the design of more potent and specific compounds for RNA targeting. Figure 10 illustrates the strong intercalation of RNA by H3BTB.

### 3.7. Molecular Dynamics Simulation

In the present study, molecular dynamics simulations were carried out to investigate the stability of the protein–ligand complex (caspase-3–H3BTB). These simulations aimed to determine the molecular interactions between the protein and the ligand and to evaluate the impact of these interactions on the stability of the complex. The protein–ligand complexes were modeled using Desmond software and were subjected to various thermal and mechanical perturbations to assess their stability. The results of these simulations were then analyzed in detail to identify key residues involved in the formation of the complex and to determine the nature of the interactions between the protein and the ligand.

**Figure 11:**
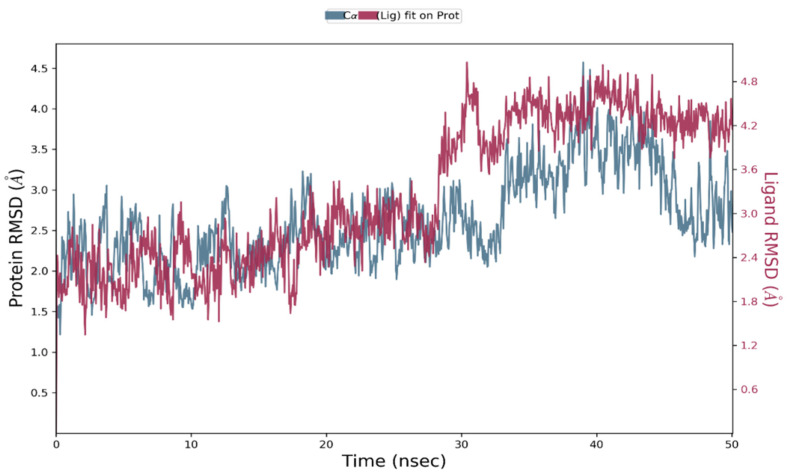
Illustration of RMSD pattern for the protein and protein–ligand complex.

The stability of apoprotein and the protein–ligand complex was investigated through the evolution of the RMSD pattern. It was notable that apoprotein demonstrated a significant stability pattern during MD simulation studies. Slight rearrangements were observed after 30 ns which became stable and equilibrated after a few nanoseconds. The observed fluctuations in the protein RMSD plot after 30 ns may be attributed to the inherent flexibility of the protein structure. While the protein structure is generally stable and well-defined, some regions of the protein may exhibit flexibility or conformational changes in response to changes in the environment or binding of ligands. This flexibility can result in fluctuations in the RMSD plot, especially in the absence of constraints or external factors. It is also worth noting that the observed fluctuations were relatively small, and the protein structure quickly returned to equilibrium, suggesting that these fluctuations did not significantly affect the overall stability of the system. In terms of protein–ligand complex stability, it was notable that the liganded protein was stable up to 30 ns after which the ligand exhibited slight fluctuations inside the active pocket. These rearrangements were short-duration and became stable after a few ns of MD simulation. The average RMSD of liganded protein was 2.8 angstroms. It was observed that the stability of the protein–ligand complex was highly dependent on the strength of the interactions between the protein and the ligand. The simulations revealed that hydrogen bonding and hydrophobic interactions were the main contributors to the stability of the complex. Hydrogen bonding interactions were found to be particularly important for the formation of stable complexes, with the hydrogen bonding residues playing a crucial role in maintaining the structural integrity of the complex. Hydrophobic interactions, on the other hand, were observed to contribute to the stability of the complex by reducing the exposure of hydrophobic residues in the protein and the ligand. Figure 11 illustrates the evolution of RMSD for the protein and protein–ligand complex.

**Figure 12:**
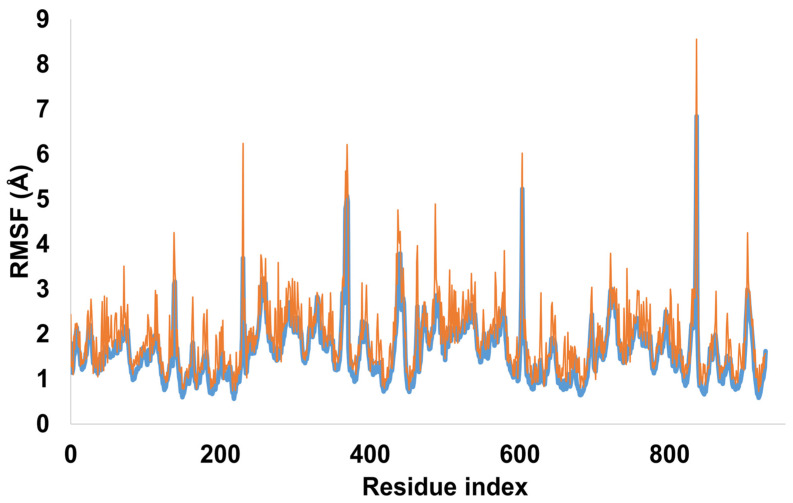
RMSF pattern for C alpha atoms of caspase-3 (orange peaks) and caspase-3–H3BTB complex illustrated by blue colored peaks.

The root mean square fluctuation (RMSF) analysis of the protein–ligand complex demonstrated its stability over the simulation time course. The RMSF values were calculated to assess the deviation of each amino acid residue from its average position, and the results showed that the protein maintained its structural integrity throughout the simulation. The RMSF analysis confirmed the robustness of the protein–ligand complex and supported the conclusion that the complex was thermodynamically stable. This stability is critical for the success of the complex in biological applications, and the RMSF analysis provided valuable insight into the stability of the protein–ligand interaction. The RMSF analysis revealed that both the apo and liganded proteins displayed stability in their respective structures. Notably, the liganded protein exhibited fewer fluctuations than the apoprotein, indicating that the binding of the ligand had a stabilizing effect on the protein structure. Additionally, several key amino acid residues, including GLY60, LYS57, MET61, THR62, SER63, ARG64, SER65, GLY165, GLU167, LEU168, ASP169, CYS170, GLY171, GLU173, TYR203, and TYR204, remained in contact with the ligand throughout the simulation. Interestingly, these residues displayed slightly higher RMSF values in the apoprotein, while their RMSF values decreased in the liganded protein, suggesting that the binding of the ligand helped to reduce the fluctuations in these key amino acid residues. In addition, important amino acid residues attached to ligands demonstrated significant stability. The average RMSF value for caspase-3 was 2.5 angstroms which is quite acceptable. The results of this study have implications for the design of new drugs and therapies targeting proteins in biological systems, as a stable protein–ligand complex is a crucial requirement for the effective functioning of drugs. Figure 12 depicts the RMSF value for the targeted complex.

**Figure 13:**
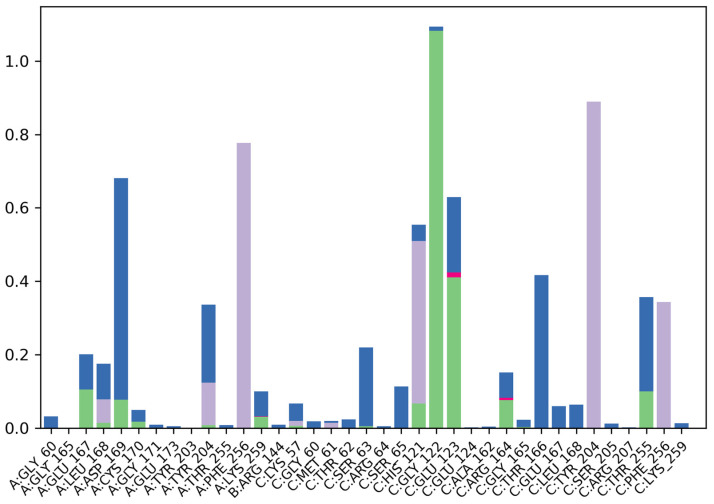
Molecular interactions observed during MD simulations, the y-axis is depicting the percentage of interaction fraction.

The stability of the protein–ligand complex was indicative of robust hydrogen bonding and hydrophobic interactions. Figure 13 highlights the potential hydrophilic and hydrophobic interactions of H3BTB with caspase-3. These interactions were observed to last for 10% to 100% of the simulated trajectory. Specifically, hydrogen bonding occurred between the compound and GLU167, ASP169, HIS121, GLY122, GLU123, ARG164, and THR255. Notably, the hydrogen bond with GLY122 lasted for the entire simulated trajectory, whereas GLU123 persisted for 40% of the simulation time. Although other amino acid residues were engaged in interactions for less than 20% of the simulated trajectory, they nevertheless played a role in stabilizing the protein–ligand complex. Regarding hydrophobic interactions, TYR204, PHE256, HIS121, and PHE356 were involved. The interactions with PHE256, HIS121, TYR204, and PHE356 lasted for 80%, 40%, 90%, and 40% of the simulation time, respectively. Additionally, several water bridges and cationic interactions contributed to the stability of the protein–ligand complex. The intricate details of the molecular interactions are illustrated in Figure 13.

**Figure 14:**
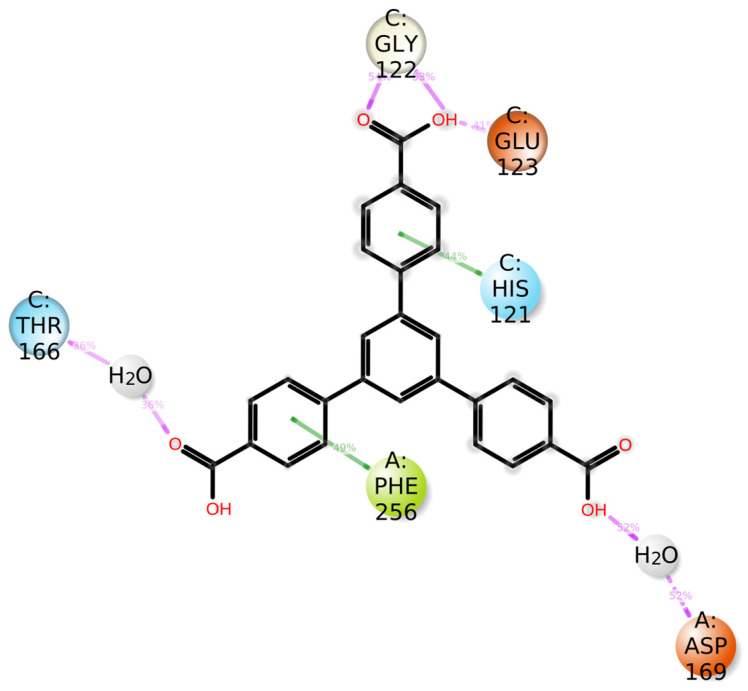
Molecular interactions observed during MD simulations.

During the course of molecular dynamics simulations, it was observed that the carboxylic group of compound a formed a conventional hydrogen bond with GLY122, which lasted for the majority of the simulated trajectory. Meanwhile, the aromatic ring of compound a exhibited a pi–pi interaction with PHE56 for more than 20% of the simulation time, contributing to the overall stability of the protein–ligand complex. Furthermore, HIS121, THR166, ASP169, and GLU123 were identified as key amino acid residues that formed hydrophobic and hydrophilic interactions with the hydroxyl group of compound a for more than 30% of the simulated trajectory. These interactions were important for maintaining the stability of the protein-ligand complex, as they helped to anchor the ligand within the binding site and promote favorable interactions between the ligand and the protein. In addition, several water bridges and cationic interactions were observed between the ligand and the protein, which also contributed to the overall stability of the complex. Specifically, water molecules were observed to bridge between the hydroxyl group of compound a and nearby amino acid residues, including GLU123 and ASP169, forming stable hydrogen bonds. Furthermore, cationic interactions were observed between the positively charged amino acid residues, such as ARG164 and LYS223, and the negatively charged carboxylic group of compound a. Overall, the detailed interaction profile presented in Figure 14 highlights the diverse array of interactions that contribute to the stability of the protein–ligand complex, including conventional hydrogen bonding, pi–pi interactions, hydrophobic and hydrophilic interactions, water bridges, and cationic interactions.

**Figure 15:**
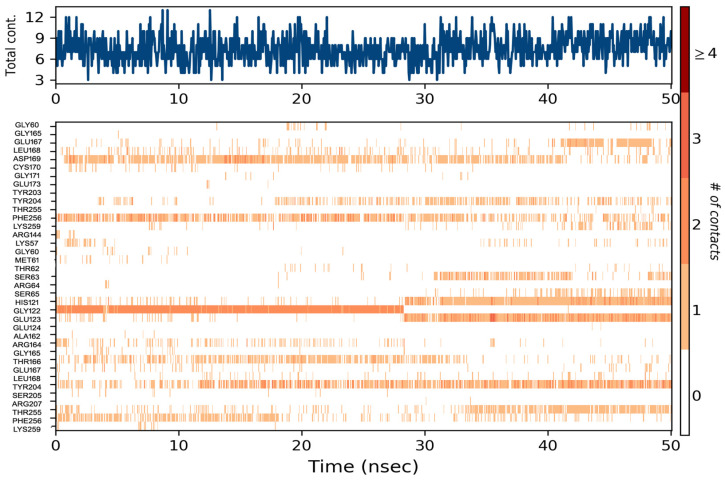
Interaction fraction observed during the simulated trajectory.

The analysis of the degree of buriedness of the compound with the targeted amino acid residues revealed interesting insights into the binding mechanism. As depicted in Figure 15, GLY122 was found to be strongly buried by compound a throughout the simulation. Moreover, TYR204, PHE256, ASP169, HIS121, and GLY123 were also buried by compound a for more than 50% of simulated trajectory. On the other hand, some amino acid residues showed a lower degree of buriedness, indicating fewer or no interactions with the ligand. It is noteworthy that the degree of buriedness may vary during the simulation due to the protein’s conformational changes and the ligand’s dynamic behavior.

## 4. Conclusions

In conclusion, this study aimed at evaluating the potential of H3BTB as an inhibitor of tumor growth through its interactions with cancer proteins, caspase-3, NF-κB, p53, and DNA. Docking simulations showed that H3BTB produced strong interactions with the proteins and DNA, with favorable binding scores and hydrogen bonding residues. The findings from FMO analysis indicated that H3BTB had a high electron affinity, particularly towards electronegative oxygen atoms and aromatic rings. Additionally, this study found that H3BTB was able to strongly intercalate with DNA, suggesting its potential as a DNA intercalator. The results of the molecular dynamics simulations confirmed the stability of the protein–ligand complex, and the RMSF analysis further supported its stability. These findings suggest that H3BTB has potential as a promising anti-cancer agent and may warrant further investigation.

## Figures and Tables

**Table 1:** Cytotoxic activity of H3BTB against human HeLa, MDA-MB-231, and MCF-7 cancer cell lines.

Compound	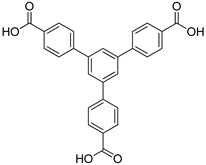			
	HeLa	MDA-MB231	MCF-7	Vero Cells
	GI_50_ ± SEM			% Growth Inhibition
H3BTB	16.2 ± 1.02	7.62 ± 0.91	9.03 ± 0.18	7.31%
Doxorubicin	4.21 ± 0.22	6.82 ± 0.59	7.32 ± 0.81	10.2%
Cisplatin	2.64 ± 0.13	2.27 ± 0.24	4.63 ± 0.21	6.67%

**Table 2:** Optimization energy, polarizability, and dipole moment of H3BTB.

Compound	Optimization Energy (Hartree)	Polarizability a.u (α)	Dipole Moment (debye)	Potential Ionization I (eV)	Affinity A (eV)	Electron Donating Power (ω−)	Electron Accepting Power (ω+)	Electro Philicity (Δω±)
H3BTB	−1490.693	326.515	4.487	0.257	0.085	0.096	0.267	0.362

**Table 3:** Global and local reactivity descriptors of H3BTB.

Compound	E_HOMO_ (eV)	E_LUMO_ (eV)	∆E_gap_ (eV)	Chemical Hardness (η)	Chemical Potential (μ)	Electrophilicity Index (ω)	Chemical Softness (S)	Electronegativity (X)
H3BTB	−0.257	−0.085	0.171	0.086	−0.171	0.170	5.821	0.171

**Table 4:** The molecular interactions of H3BTB.

Complex	Docking Score (kcal/mol)	Hydrogen Bonding Residues	Hydrogen Bond Length (Angstroms)	Hydrophobic Interactions Residues
Caspase-3–H3BTB	−10.4	Gly60	2.88	Cys170, Leu168, Thr255, His121, Tyr204, Gly122, Gly165, Thr166, Phe256, Leu168, Thr166, Phe256, and Thr62
NF-κB–H3BTB	−8.8	Phe239, Phe239, Gln266, and Thr257	3.02, 2.94, 3.14, and 3.21	Gly259, Trp258, Gln241, Arg260, Ser238, Gly237, Glu222, and Lys28
P53–H3BTB	−8.1	Gln23, Gln23, Ser204, and Ala200	2.94, 3.03, 2.80, and 2.77	Arg203, Leu125, Pro115, Tyr92, Ile22, Cys114, and Arg104
DNA–H3BTB	−8.3	Dc11	3.14	Gd16, Da17, Dg10, Dc9, Dt19, Dt8, and Da18
RNA–H3BTB	−9.5	C146, C147, U77, U145, G127, G126, G76, and G78	2.98, 2.85, 2.73, 3.22, 2.71, 3.21, 3.20, and 2.73	G149, G78, and A150

## Data Availability

Data will be available on request to corresponding author.
